# Time for Radical Changes in Brain Stem Nomenclature—Applying the Lessons From Developmental Gene Patterns

**DOI:** 10.3389/fnana.2019.00010

**Published:** 2019-02-12

**Authors:** Charles Watson, Caitlin Bartholomaeus, Luis Puelles

**Affiliations:** ^1^School of Biological Sciences, University of Western Australia, Perth, WA, Australia; ^2^Neuroscience Research Australia, The University of New South Wales, Sydney, NSW, Australia; ^3^Department of Human Anatomy and IMIB-Arrixaca Institute, School of Medicine, University of Murcia, Murcia, Spain

**Keywords:** brain stem, hindbrain, midbrain, isthmus, rhombomeres

## Abstract

The traditional subdivision of the brain stem into midbrain, pons, and medulla oblongata is based purely on the external appearance of the human brain stem. There is an urgent need to update the names of brain stem structures to be consistent with the discovery of rhomobomeric segmentation based on gene expression. The most important mistakes are the belief that the pons occupies the upper half of the hindbrain, the failure to recognize the isthmus as the first segment of the hindbrain, and the mistaken inclusion of diencephalic structures in the midbrain. The new nomenclature will apply to all mammals. This essay recommends a new brain stem nomenclature based on developmental gene expression, progeny analysis, and fate mapping. In addition, we have made comment on the names given to a number of internal brain stem structures and have offered alternatives where necessary.

## Introduction

For over a century, teachers and scientists have described the mammalian brain stem as having three parts—the midbrain, the pons, and the medulla oblongata—and the names of numerous structures inside the brain stem are consistent with this subdivision. This subdivision was based purely on the external appearance of the human brain stem and there is an urgent need to update the names of brain stem structures to be consistent with modern research findings relative to molecularly defined brain stem developmental units. Studies of developmental gene expression show that the current use of the term “pons” is in most cases very misleading (Puelles et al., 2013; Watson et al., 2017a). In addition, gross misinterpretations of brain stem organization have led to the mistaken inclusion of diencephalic structures in the midbrain, and the failure to recognize the isthmus as the first segment of the hindbrain. This essay will summarize the problems that have arisen from the conventional use of the traditional brain stem nomenclature, and will suggest alternatives based on developmental gene expression, progeny analysis, and fate mapping. In addition, we will comment on the names given to a number of internal brain stem structures and offer alternatives where we think it necessary.

The key to understanding the “natural” (i.e., gene-modulated) anatomy of the brain stem lies in an appreciation of its segmental rostrocaudal organization, without forgetting its parallel dorsoventral differentiation. A complete picture of the segmental organization has been revealed by a number of studies of gene expression during development, which have been summarized by Puelles et al. (2013) and Tomás-Roca et al. (2016).

## Gene Expression Reveals the Segmental Organization of the Brain Stem

The segmental organization of the brain stem was first observed by embryologists in the late nineteenth century, who described a series of outpouchings in the developing vertebrate brain stem (von Baer, 1828; Orr, 1887). The significance of this finding was lost in the subsequent period dominated by the columnar organization theories of Herrick (1910, 1948). But over about the past 25 years, the outpouchings have been recognized as evidence for the fundamental segmental organization of the brain stem. The change came about through the advent of studies on developmental gene expression (e.g., Gaunt et al., 1986; Murphy et al., 1989; Wilkinson et al., 1989a,b; Sundin and Eichele, 1990; Krumlauf et al., 1993), the creation of molecularly-defined regional progeny, and clonal restriction (Lumsden and Keynes, 1989; Fraser et al., 1990; Lumsden, 1990, 1991). These gene-based progeny studies were enabled by the invention of gene targeting in mice (Capecchi, 1989). It is now clear that the brain stem of all vertebrates is made up of a rostro-caudal series of segments that arise in early development and impose an anatomical and functional organization that persists in the adult brain. An additional point of significance is that the midbrain has in recent years been ascribed to the forebrain, taking it out of the brain stem. The midbrain has been found to share a number of gene expression patterns with diencephalon and hypothalamus and lacks true continuity with the hindbrain (Puelles, 2013). The midbrain contains two segments, called mesomeres (Puelles et al., 2012a; Puelles, 2013), whereas the hindbrain is divided into 12 neuromeres—the isthmus and 11 rhombomeres (Puelles et al., 2013; Tomás-Roca et al., 2016; Watson et al., 2017a). Unfortunately, some authors (notably those led by Lumsden and Krumlauf) have consistently ignored the significance of the isthmus and have not accepted the existence of the four caudal rhombomeres (r8 to r11), based on the fact that they lack overt constrictions between them (e.g., Lumsden and Krumlauf, 1996; Tümpel et al., 2009). However, the gene expression evidence for the isthmic segment (Watson et al., 2017c) and the presence of four “hidden” rhombomeres, known as cryptorhombomeres, is now very strong (Marín et al., 2008; Puelles, 2013; Puelles et al., 2013; Tomás-Roca et al., 2016). One surprising finding in relation to the caudal rhombomeres is that the pyramidal decussation is located in the spinal cord, and not in the caudal hindbrain as has been traditionally assumed (Tomás-Roca et al., 2016). The pyramidal tract fibers decussate after they cross the medullo-spinal boundary and so the pyramidal decussation in no longer a component of the hindbrain.

The first comprehensive attempt to illustrate the boundaries and contents of the segmental elements of the brain stem (two mesomeres, isthmus, and 11 rhombomeres) in different planes of section was presented in the chick brain atlas of Puelles et al. (2007). Many of the segments in the brain stem in birds and mammals can be confidently identified by the presence of one or more signature nuclei; examples are the trochlear nucleus in the isthmus and the abducens nucleus in r5. A diagram summarizing mammalian segmental components can be found in Tomás-Roca et al. (2016), and a modified version of this figure is shown in our **Figure 4**. Table 1 shows the segmental position of selected structures in the mammalian brain stem and adjacent diencephalon and spinal cord.

**Table 1:** Segmental components of the mammalian caudal diencephalon, midbrain, and hindbrain and position of major structures within these segments.

**DIENCEPHALON**		
**Diencephalic prosomere 1 (dp1)**		
	Posterior commissure	pc
	Pretectal nuclei	PT
	Darkschewitsch nucleus	Dk
	Interstitial nucleus of Cajal	InC
	Red nucleus, parvocellular part	RPC
**MIDBRAIN**		
Mesomere 1 (m1)	Superior colliculus	SC
	Inferior colliculus	IC
	Oculomotor nucleus	3N
	Emerging oculomotor nerve	3n
	Red nucleus, magnocellular part	RMC
Mesomere 2 (m2)	Sagulum nucleus	Sag
	Retrorubral field (DA8)	RRF
	Subbrachial nucleus	SubB
**HINDBRAIN**		
**Isthmocerebellar (prepontine)**		
Isthmus (is)	Trochlear nucleus	4N
	Emerging trochlear nerve	4n
	Parabigeminal nucleus	PBG
	Microcellular tegmental nucleus	MiTg
	Prodomal interpeduncular nucleus	IPpro
Rhombomere 1 (r1)	Locus coeruleus	LC
	Rostral interpeduncular nucleus	IPR
	Caudal interpeduncular nucleus	IPC
	Parabrachial nuclei	MPB/LPB
**PONTINE REGION**		
Rhombomere 2 (r2)	Rostral motor trigeminal nucleus	5N
	Emerging motor trigeminal nerve	5n
Rhombomere 3 (r3)	Caudal motor trigeminal nucleus	5N
	Rostral pontine nuclei	Pn
Rhombomere 4 (r4)	Emerging facial nerve	7n
	Caudal pontine nuclei	Pn
**RETROPONTINE**		
Rhombomere 5 (r5)	Abducens nucleus	6N
	Emerging abducens nucleus	6n
	Superior olive and trapezoid body	SOl/tz
Rhombomere 6 (r6)	Facial nucleus (migrated)	7N
	Emerging glossopharyngeal nerve	9n
**MEDULLA OBLONGATA**		
Rhombomere 7 (r7)	Compact ambiguus nucleus	AmbC
Rhombomere 8 (r8)	Compact ambiguus nucleus	AmbC
	Rostral inferior olive	IO
Rhombomere 9 (r9)	Semicompact ambiguus nucleus	AmbSC
	Middle inferior olive	IO
Rhombomere 10 (r10)	Loose ambiguus nucleus	AmbL
	Caudal inferior olive	IO
	Area postrema	AP
Rhombomere 11 (r11)	Retroambiguus nucleus	RAmb
**ROSTRAL SPINAL CORD**		
C1 segment	Pyramidal decussation	pyx

**Figure 1:**
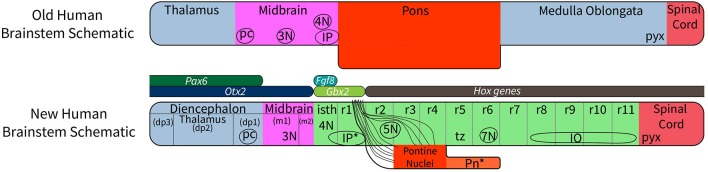
A diagram to compare the traditional view of subdivisions of the human brain stem with the new system of segmentation revealed by developmental gene expression. At the top, the subdivisions of the “old” human brain stem (the traditional version) are based on the assumption that the midbrain extends from the thalamus to the rostral margin of the pons; this concept wrongly holds that the pretectum (dp1) and the isthmus (isth) belong to the midbrain (Puelles et al., 2012a). Comparing the traditional version of the human brain stem with the new segmental schema (bottom schema) we see that the “old” pons was held to extend between levels r1 to r6. In reality, r5 and r6 represent a hidden rostral retropontine part of the “medulla oblongata,” whereas the migrated basilar pons is located only within r3 and r4. Part of the confusion relating to the extent of the pons is due to a mushroom-like rostral expansion of the pons created by rostral pontine cerebellopetal fibers that surround the trigeminal root in r2 as they approach the cerebellum in r1(see Figure 6), thus adding part of r2 to the apparent pontine bulge in humans. On the other hand, mammals with less massive pontine development than humans show a simpler, less deformed general arrangement, which leaves the ventral surface of r5 and r6 exposed. In addition, the “old” version of the human brain stem places the pyramidal decussation (pyx) at the caudal end of the medullary brain stem, whereas the decussation actually lies in the rostral spinal cord. The most important difference between the “new” human brain stem and the generic mammalian brain stem is that the basilar pons in the human bulges rostrally into r2, where only crossed fibers of the middle cerebellar peduncle are found, and caudally, where the overhanging part of the basilar pontine nuclei partly hides the underlying rhombomeres r5 to r6 (Pn^*^). The positions of the oculomotor (3N), trochlear (4N), and facial (7N) nerve nuclei, the interpeduncular nuclei (the prodromal, caudal and rostral IP parts are collectively labeled as IP^*^); the posterior commissure (pc), and the inferior olive are shown for reference. The rostrocaudal extent of key developmental genes is shown in the middle of the diagram. Note *Fgf8* codes for the morphogen signal of the isthmic organizer, whose hindbrain gradient ends at the r1/r2 boundary. This image is loosely based on a figure presented by Watson et al. (2017a).

**Figure 2:**
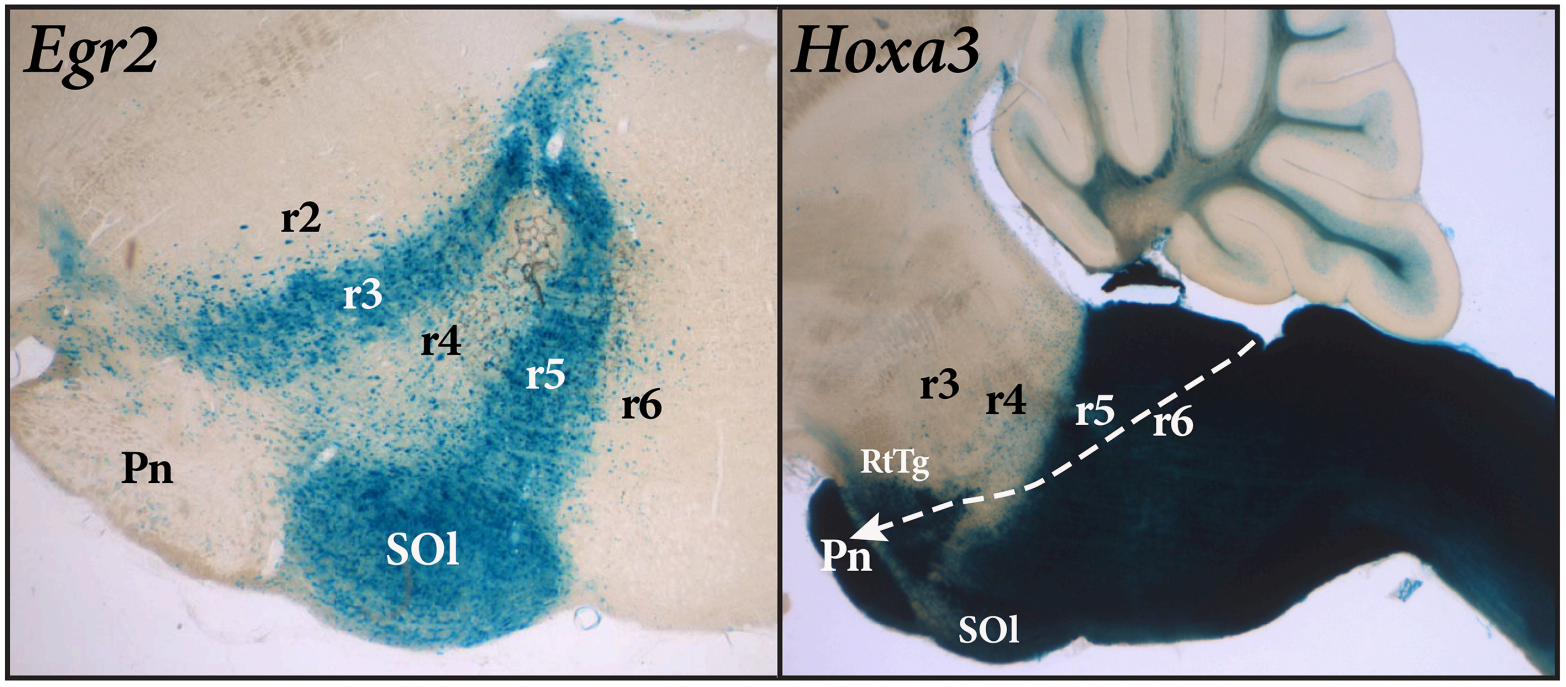
Sagittal sections of mouse brain stem with *Egr2*-Cre and *Hoxa3*-Cre fate mapping. The blue *Egr2* label is seen in the cells of rhombomere 3 (r3) and rhombomere 5 (r5). Rhombomere 5 contains the labeled cells of the superior olive (SOl), but the pontine nuclei within r4 (Pn), which migrate from r6-r7, remain largely unlabeled. The section on the right, showing expression of *Hoxa3*, reveals a sharp delineation between rhombomere 4 (r4) and rhombomere 5 (r5). However, the pontine nuclei within r3 and r4, as well as the RtTg nucleus, are labeled in this case because they have migrated from the rhomic lip of rhombomeres 6 and 7 (r6-r7) as indicated by the path of the white arrow.

A relatively small set of genes is involved in establishing the rostrocaudal segmental plan of the central nervous system. Those vital to brain stem development include *Pax* family genes, *Otx2, Wnt1, Gbx2, Fgf8, Shh genes*, and *Hox* family genes. Their role in the segmentation of the brain stem is summarized in Figure 1, which shows that expression of *Pax 6* in the alar diencephalon ends sharply at the junction between the pretectal area and the midbrain (Schwarz et al., 1999; see images in Puelles et al., 2012a; Duan et al., 2013), *Otx2* is expressed in forebrain and midbrain (Puelles et al., 2012a,b); *Gbx2* is expressed in the rostral hindbrain (isthmus and r1) but not in the midbrain (Puelles et al., 2012a); *Fgf8* is selectively expressed in the isthmus (Watson et al., 2017c); and the *Hox* genes are expressed from r2 to the caudal end of the spinal cord (Puelles et al., 2013). The expression of the *Hox-*related gene *Egr2* reveals the anatomy of rhombomeres 3, 4, and 5 in a convincing way (Figure 2).

There is a question as to whether the gene expression data acquired from mice can be confidently applied to other mammals, and perhaps to other vertebrates. We are confident such extrapolations can be made, because the anatomy and development of the brain stem is highly conserved (for a general discussion of this issue see Nieuwenhuys et al., 1998; Gilland and Baker, 2005). For example, the pattern of gene expression in the development of the brain stem in chicks mirrors that described in the mouse in almost every respect, even though the species are separated by around 300 million years of evolution. A few exceptions do exist (such as the translocation of the facial motor nucleus from r4 to r6 in mammals), but the point to point similarities are extraordinary (Cambronero and Puelles, 2000; Puelles et al., 2007; Tomás-Roca et al., 2016). However, the evolutionary history of brain stem development is a much bigger subject than we have attempted to address in the present paper.

## Problems With Traditional Brain Stem Nomenclature

When the traditional nomenclature of the brain stem is tested against the new understanding of brain stem organization based on developmental gene expression, five major areas of misinterpretation become apparent. These are the true identity of the pons, the existence of the isthmus, the true definition of the midbrain without diencephalic and hindbrain additions, the location of the substantia nigra and VTA (though this is rather a diencephalon problem), and the segmental origin of the cerebellum.

### The True Identity of the Pons

The primary problem with the use of the word “pons” is that its historical meaning attaches to the voluminous formation seen on the ventral surface of the human brain. The basilar pontine formation is exceptionally large in humans (correlative with expansion of the cerebral cortex), and this has led to misinterpretation over its true topological position. In many mammals, the basilar pontine nuclei (Pn) and the reticulotegmental nucleus (RtTg) aggregate at the ventral part of rhombomeres 3 and 4, and the pontine bulge is restricted to the ventral surface of these two rhombomeres. An interesting developmental feature of the basilar pons is that the neurons that form the pontine nuclei develop in the rhombic lip of rhombomeres 6 and 7 and then migrate tangentially under the pia to their final location in rhombomeres 3 and 4 (Figure 2).

**Figure 3:**
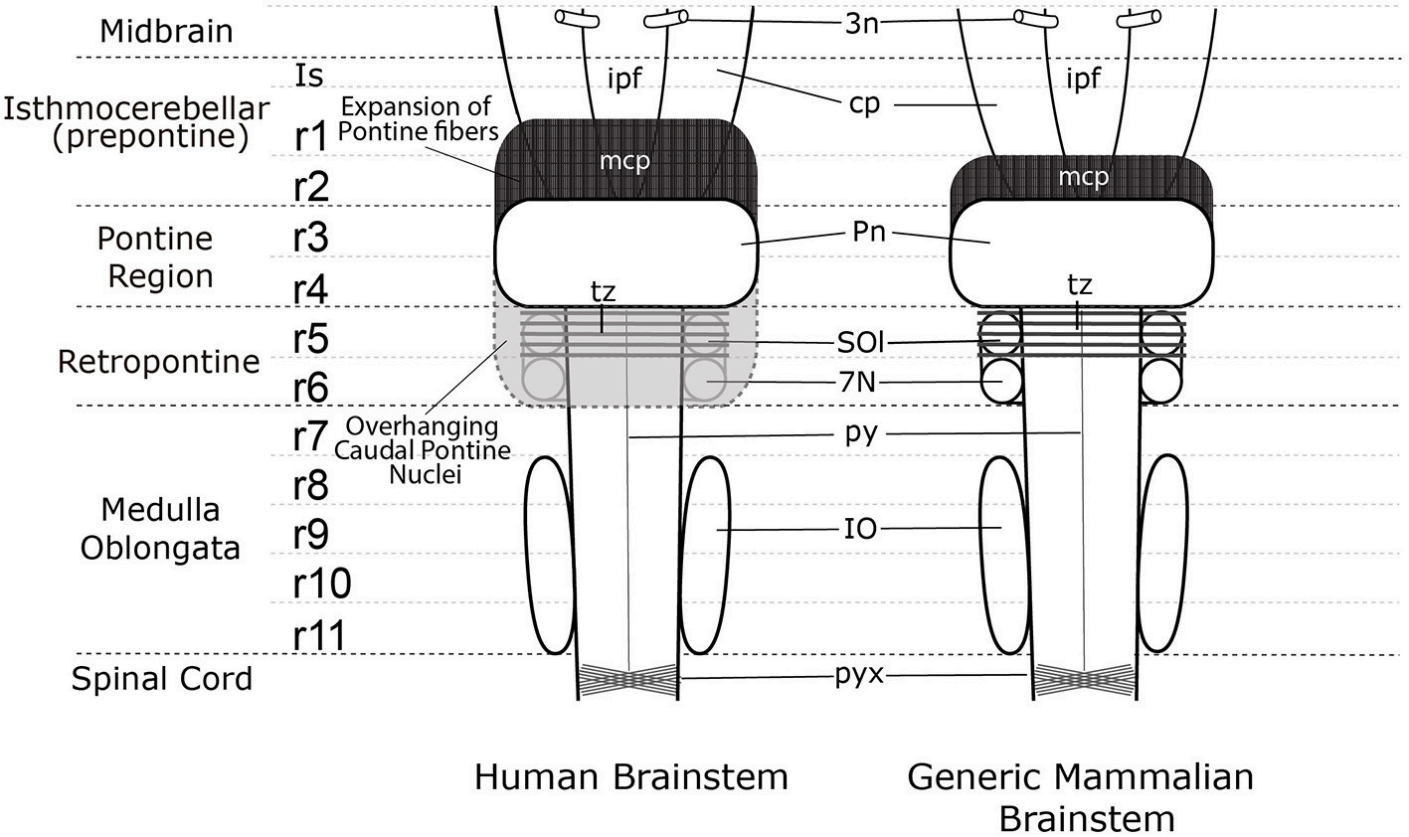
A comparison of the external view of the human brain stem (left) and generic mammalian brain stem (right). In the midbrain the emerging oculomotor nerve (3n) is shown. Note the interpeduncular fossa extends into the prepontine hindbrain (ipf), where the interpeduncular nuclear complex is found (not shown). The surfaces of the cerebral peduncles (cp) and the interpeduncular fossa (ipf) visible in the human brain stem are reduced by the rostral expansion of the cerebellopetal pontine fibers coursing through r2 into the cerebellum in r1(middle cerebellar peduncle—mcp). The trapezoid body (tz) and superior olive (SOl) identify rhombomere 5 (r5), but these structures are not visible on the ventral surface of the human brain stem as they are covered by the overhanging caudal pons. The migrated facial nucleus (7N) is found in rhombomere 6 (r6) (Di Bonito et al., 2013; Puelles et al., 2018), but it is also covered by the overhanging caudal expansion of pontine nuclei in the human brain stem. The inferior olive extends from rhombomere 8 (r8) to rhombomere 11 (r11). The spinal cord begins at the start of the pyramidal decussation (pyx).

**Figure 4:**
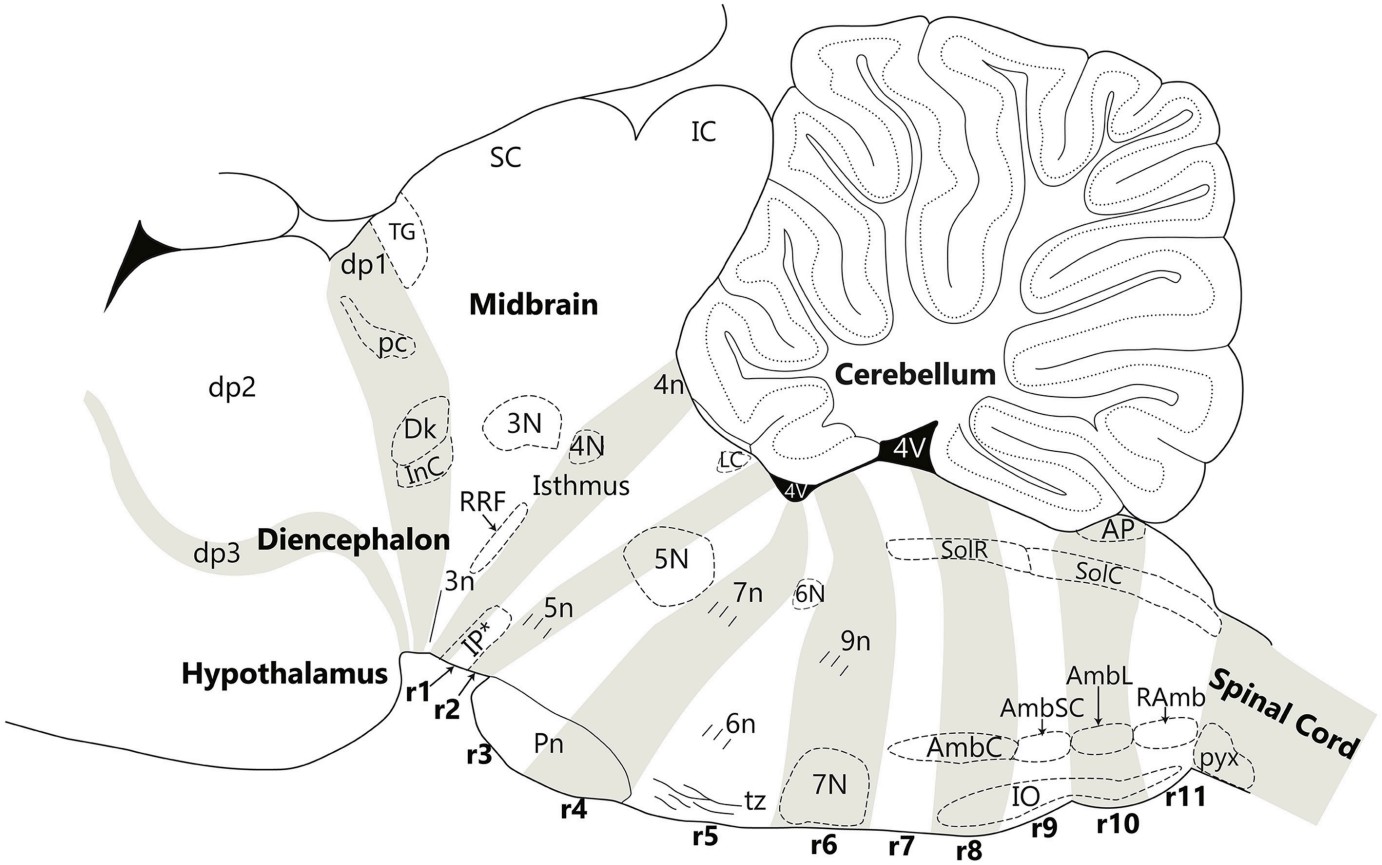
Nuclear and fiber landmarks that identify the segments of the hindbrain, midbrain and diencephalon. In this Figure, the cerebellum, fourth ventricle and hypothalamus are labeled for orientation. Note that fate-mapping data have shown that the cerebellum is a tectal structure restricted to the isthmus and r1, irrespective that in the adult it overhangs far backwards over the dorsal choroidal surface of the pontine, retropontine and medullary regions. The diencephalic prosomere 1 (dp1), which contains the pretectal posterior commissure (pc), Darkschewitsch nucleus (Dk) and the interstitial nucleus of Cajal (InC), is delimited anteroposteriorly by the extent of the posterior commissure (pc). The midbrain contains the oculomotor nucleus (3N) and emerging oculomotor nerve (3n) in mesomere 1 and the retrorubral field (RRF) in mesomere 2. Mesomere 2 is a thin wedge of the midbrain, caudal to 3N, the red nucleus and the inferior colliculus. The hindbrain is comprised of twelve segments—the isthmus (r0) and rhombomeres 1–11 (r1 to r11). The isthmus contains the trochlear nucleus (4N), the emerging trochlear nerve (4n) and the prodromal part of the interpeduncular nucleus (IP^*^). Rhombomere 1 (r1) contains the rostral and caudal parts of the interpeduncular nucleus (IP^*^), the dorsal and ventral tegmental nuclei, and the locus coeruleus (LC). Rhombomere 2 (r2) contains the rostral part of the motor trigeminal nucleus (5N) and the emerging motor trigeminal nerve. Rhombomere 3 (r3) contains the caudal part of the motor trigeminal nucleus (5N) and the rostral pontine nuclei (Pn). Rhombomere 4 (r4) contains the caudal pontine nuclei (Pn) and the emerging facial nerve (7n). Rhombomere 5 (r5) contains the abducens nucleus (6N), the emerging abducens nerve (6n), and the decussation of the trapezoid body (tz), along with the superior olivary complex. Rhombomere 6 (r6) contains the migrated facial nucleus (7N) and the emerging glossopharyngeal nerve (9n). Rhombomere 7 (r7) and 8 (r8) contain the compact ambiguus nucleus (AmbC) and the rostral end of the solitary nucleus (gustatory nucleus—SolR). Rhombomere 8 also contains the rostral tip of the inferior olive (IO). Rhombomere 9 (r9) contains the semicompact ambiguus nucleus (AmbSC) and the middle region of the inferior olive (IO). Rhombomere 10 (r10) contains the loose ambiguus nucleus (AmbL), the area postrema (AP), and the caudal region of the inferior olive (IO). Rhombomere 11 (r11) contains the retroambiguus nucleus (RAmb) and the caudal tip of the inferior olive. The spinal cord begins at the start of the pyramidal decussation (pyx).

On the other hand, human anatomy textbooks uniformly state that the pons extends from the *caudal end* of the midbrain to the *beginning* of the medulla oblongata just rostral to the exit of the vestibulocochlear and abducens nerves. The differential growth of the basilar pons in humans hides much of the rostral prepontine hindbrain (from isthmus to part of rhombomere 2), on one side, and the part of the retropontine hindbrain containing the abducens nucleus, superior olive, and facial nucleus, on the other (Figures 3, 4).

One result of the superimposition of the human version of pontine topography and nomenclature to those mammals with a small basilar pons is that many structures far away from the basilar pons are called “pontine” because in the human brain they are overlaid by the enlarged “pontine” region. The solution to this problem is relatively simple: discontinue the use of the word “pons” as a topographical descriptor in all mammals, and restrict the use of the term pons to the basilar pontine formation in r3-r4. Note the variable pontine “expansion” into r1 and r2 in primate brains lacks any basilar pontine nuclei (Pn) in its interior, and contains exclusively crossed fibers of the middle cerebellar peduncle (mcp) that surround the trigeminal root in alar r2 (see **Figure 6**). The modern segmented hindbrain model emphasizes the need to distinguish prepontine, pontine, retropontine and medullary territories, each of which appears subdivided into transversal rhombomeric domains. This provides a new level of precision to support modern anatomical and functional analysis.

### The Existence of the Isthmus

The isthmus, understood as a distinct hindbrain segment separating the midbrain from the other hindbrain rhombomeres, was already identified morphologically by His (1893, 1895), but was later arbitrarily ascribed to the midbrain in conventional neuroanatomical texts. In contemporary works, the isthmic territory is defined early in development by the selective expression of *Fgf8* (coding for the diffusible morphogen FGF8, which serves as the signal of the isthmic organizer—signal needed for the formation of the cerebellum and the caudal midbrain). The mature progeny of the isthmus have been demonstrated in a recent Cre *Fgf8* lineage study (Watson et al., 2017c). Within the isthmic territory so defined, lie the trochlear nucleus (and its emerging nerve), the parabigeminal nucleus, the microcellular tegmental nucleus, and the decussation of the superior cerebellar peduncle (Watson et al., 2017c). The isthmus therefore lies between the caudal midbrain and rhombomere 1 (r1).

Most neuroanatomical texts used by health science students do not comment on the presence of the isthmus at all (e.g., Hendelman and Walter, 2005; Haines, 2012; Jacobson et al., 2017; Mtui et al., 2017). A few make note of the organizing role of the isthmic region in the development of the midbrain/hindbrain junction, but do not acknowledge its presence in the mature brain (e.g., Martin, 2003; Nieuwenhuys et al., 2008; Barker et al., 2017). A small number of textbooks recognize the presence of the isthmus in both the developing and developed brain but mistakenly describe it as forming the caudal part of the midbrain (e.g., Butler and Hodos, 2005; Kiernan and Rajakumar, 2013). The modern concept of the isthmus concept establishes a new caudal boundary for the midbrain region, which coincides with the caudal expression limit of the gene *Otx2* in all vertebrates (Puelles, 2013; Puelles et al., 2013).

### The Mistaken Inclusion of Diencephalic Structures in the Rostral Midbrain and the Modern Rostral Midbrain Boundary

The diencephalon consists of three segments (diencephalic prosomeres 1, 2, and 3, labeled dp1–3 in Figure 1) defined by gene expression (Puelles et al., 2012a; Puelles, 2013). The caudal diencephalic prosomere (dp1—the pretectal region) is sharply separated from the rostral border of the midbrain by a plane passing just behind the posterior commissure and in front of the oculomotor nerve root (Figures 3, 4; Puelles et al., 2012a). Diverse developmental genoarchitectonic studies reveal that a number of caudal diencephalic structures have been mistakenly placed within the boundaries of the midbrain, while experimental analysis has shown that a midbrain fate is incompatible with some genes expressed in the diencephalic pretectum, such as *Pax6* (Puelles, 2013, 2016).

These misplaced structures include the nucleus of Darkeschewitz (dp1), the interstitial nucleus of Cajal (dp1), the rostral (parvicellular) red nucleus (dp1), the pre-Edinger-Westphal nucleus (dp1), the subcommissural organ, the posterior commissure and its related nuclei (dp1), and the medial terminal nucleus of the accessory optic tract (dp1, dp2, and dp3). Moreover, the classical “posterior pretectal nucleus” is now ascribed to the rostral midbrain (m1), since it lies in the rostral part of the superior colliculus, but caudal to the posterior commissure. This nucleus is now named the “tectal gray” (TG, see Figure 4) which is consistent with comparative usage in tetrapods (Puelles et al., 2012a).

A partial explanation for the confusion relating to the rostral and caudal boundaries of the midbrain is lack of appreciation of the impact of the cephalic flexure on giving a marked wedge shape to the midbrain. The cephalic flexure is a sharp bend of almost 180 degrees in the neural axis at the rostral end of the brain stem, so that the ventral surface of the midbrain is compressed into a very small area between the diencephalon and the isthmus, coinciding with the region containing the emerging root of the oculomotor nerve. In a sagittal section, this results in the midbrain forming a wedge shaped profile. In fact, the emerging rootlets of the oculomotor nerve provide the only reliable guide to the identification of the ventral surface of the midbrain (Figure 4; see also Puelles et al., 2012a). Traditional representations of the midbrain have arbitrarily attempted to endow it with a ventral surface of about the same extent as the dorsal (tectal) surface. Based on this error, both textbooks and journal articles placed many structures within the midbrain that actually belong to the isthmus (caudally) or diencephalon (rostrally). The correct location of many of these structures is seen in Figure 4, which shows the boundaries of the midbrain on a diagram of a sagittal section of a rodent brain.

### The Location of the Substantia Nigra and the VTA

A further complication resulting from the severe cephalic flexion of the neuraxis at the level of the midbrain is a misunderstanding of the segmental location of the substantia nigra and the VTA. It is widely assumed that both of these structures lie *within the midbrain*, but in fact only a caudal portion of both the substantia nigra and the VTA can be found in the compressed true ventral midbrain (Figure 4), and the rostral parts of the substantia nigra and VTA lie in the diencephalon, across its prosomeres 1, 2, and 3. The caudalmost parts of these dopaminergic populations lie in the isthmus (Puelles et al., 2012a,b). The overall result is that only about one quarter of the substantia nigra and VTA can be said to belong to the midbrain, and modern literature refers to a “mesodiencephalic SN/VTA complex.” Some differential gene expression has been observed along these four parts of the SN/VTA, suggesting that each segmental module possibly manifests subtle differential properties (e.g., in projection targets or afferent sources, or in sensitivity to degenerative changes in Parkinson's disease).

### The Segmental Origin of the Cerebellum

The cerebellum is an outgrowth of the dorsalmost alar plate of the caudal isthmus and the first rhombomere (Alvarez-Otero et al., 1993; Aroca and Puelles, 2006). It is therefore an integral part of the prepontine hindbrain, contradicting the old assumption that it forms a developmental unit with the pons. The vermis of the cerebellum is mainly derived from the rhombic lip of the isthmic alar plate, and the hemisphere of the cerebellum is mainly derived from the rhombic lip of the r1 alar plate, as demonstrated by experimental fate mapping and recent progeny analysis (Alvarez-Otero et al., 1993; Wingate, 2001; Aroca and Puelles, 2006; Watson et al., 2017a,b).

## Options for Renaming Parts of the Brain Stem

The study of developmental gene expression makes it clear that the hindbrain is composed of 12 segments—the isthmus (which can be counted as r0) and the other 11 rhombomeres. The reason referring to the isthmus as r0 is that the isthmus territory was long thought to develop inside r1. And once it was realized it was an independent rhombomere [in fact the first one in the series the r0 convention was adopted to avoid changing all other rhombomere numbers; (Puelles, 2013)]. Embryologists have long considered the isthmus to be a part of the hindbrain, starting from the work of His (1893, 1895), and later complemented by Palmgren (1921), Vaage (1969, 1973) and Puelles and Martinez-de-la-Torre (1987), so the concern as to whether the traditional term “rhombencephalon” includes or not the isthmus seems a moot one.

The solution is to acknowledge the existence of 12 hindbrain rhombomeres (r0 to r11) sharing a number of gene determinants and cell fates not present in the midbrain (which should now be considered to form the caudal part of the forebrain). For example, the genes which lead to the specification of serotonergic neurons are found only in rhombomeres 0 to 1 (r0 to r11), and are not generated in the midbrain. Note that the newly named r0 element is synonymous with the classic name “isthmus,” since this term consistently refers to the rostralmost part of the hindbrain or rhombencephalon. It is important to note again here that the cerebellum is a developmental dorsal alar derivative of the r0 and r1 units, and so it is also an intrinsic part of the hindbrain. Some previous uses of the term rhombencephalon apparently excluded the cerebellum. The close developmental relationship between the cerebellum and the rostral or, modernly, prepontine hindbrain is not widely appreciated, and the cerebellum is often wrongly treated as if it were an entity separate from the remainder of the brain stem.

**Figure 5:**
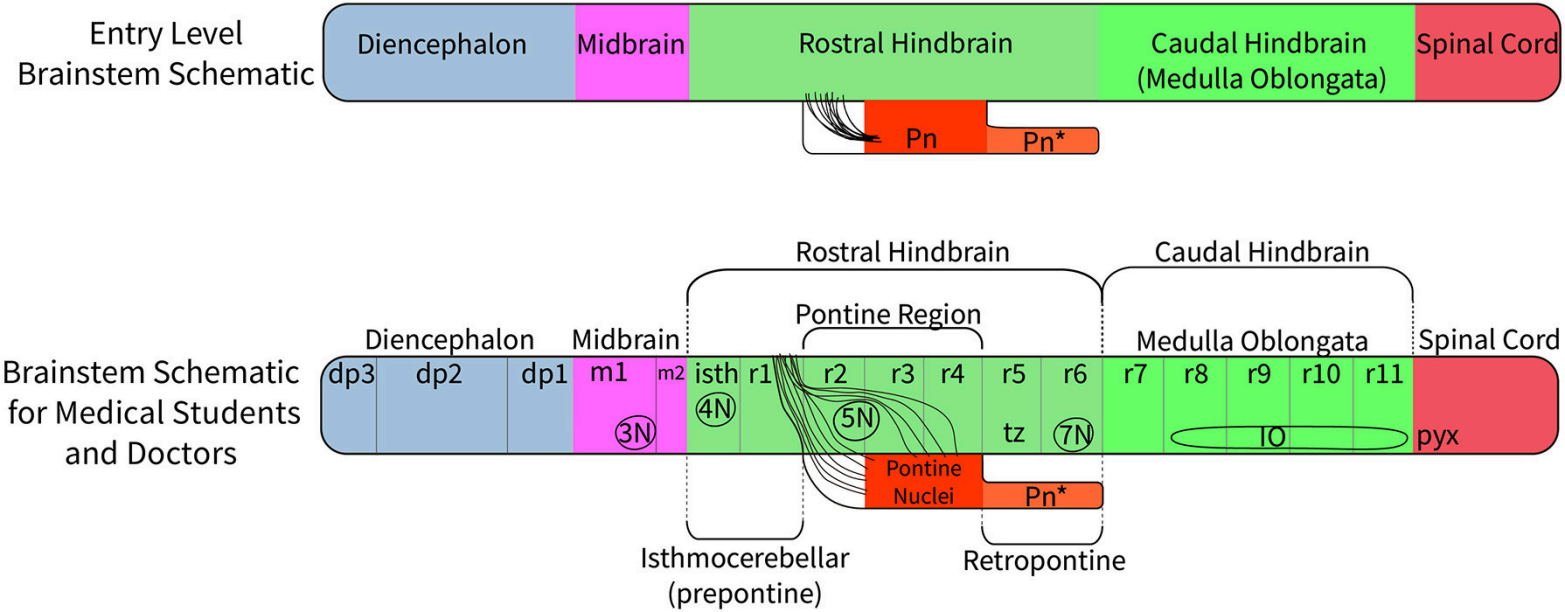
A suggested approach to represent the nomenclature for the diencephalon, midbrain and hindbrain for students at different levels of education. At an entry level (such as a high school level) a student would merely need to understand that on the basis of molecular regionalization there are rostral and caudal parts of the hindbrain. They should know that cerebellar evolutionary enlargement causes the pons (Pn) in primates to form a ventral bulge starting roughly at the middle of the rostral hindbrain, but forming a mushroom-like expansion with pontocerebellar fibers stretching forwards within neighboring rostral hindbrain areas to reach the cerebellum. In humans there is an additional pontine deformation overhanging the ventral surface of the caudalmost rostral hindbrain (Pn^*^). At a medical student and health professional level, the structures which need to be recognized include the three segments of the diencephalon (dp3, dp2, and dp1), the signature contents of the midbrain (oculomotor nucleus, 3N, and the emerging oculomotor nerve, not pictured) and the full set of hindbrain rhombomeres (isth/r0–r11). The intermediate hindbrain position of the pontine bulge at r2-r4, defines the boundaries of the prepontine (r0,r1 or isthmocerebellar) and retropontine (r5, r6) subregions. The pontine nuclei in r3 to r4 give rise to the crossed middle cerebellar peduncle which reaches forward in front and behind the trigeminal root (5n) in r2 to enter the cerebellum through r1. In humans, the caudal part of the basilar pontine nuclei overhang, and therefore hide, the most of the ventral surface of rhombomeres 5 and 6; thus r5 and r6 actually represent a distinct retropontine subregion, as a transition into the medullary region (r7 to r11).

**Figure 6:**
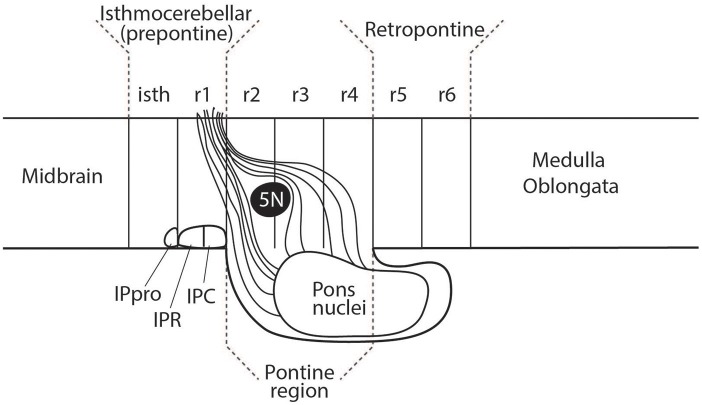
The rostral part of the interpedunclar nucleus (IPR) is often mistakenly placed in the isthmus. This diagram shows the fate-mapped true location of the prodromal interpeduncular subnucleus (IPpro) in the isthmus (isth) and the location of both the IPR and IPC subnuclei in rhombomere 1 (r1). This relates to an apparent subdivision of r1 into distinct rostral and caudal parts, a concept which has received inadequate attention (see Vaage, 1973; Alonso et al., 2012; Puelles, 2013).

There have been various attempts to harmonize or conciliate the parts of the neuromeric hindbrain with the older subdivision into pons and medulla (see Watson et al., 2017a). We suggest dividing the hindbrain into isthmocerebellar or prepontine (r0, r1), pontine (r2, r3, and r4), retropontine (r5 and r6) and medullary (r7 to r11) levels (see Figures 5, 6). These divisions provide a logical approach to naming the areas of the hindbrain associated with the pontine regions. This approach retains largely unchanged the use of the term medulla oblongata, which is common to all current textbooks. There may subsist, however, also a need for a larger scale subdivision of the hindbrain for some clinical purposes. We therefore suggest that the region from isthmus (r0) to rhombomere 6 can be referred to as “rostral hindbrain” and the region from rhombomeres 7 to 11 can be referred to as “caudal hindbrain” (or medulla oblongata) (Figure 5). This definition of the rostral hindbrain includes the isthmocerebellar, pontine and retropontine regions described above. However, we realize that in order to make embryological and physiological rhombomere-related scientific progress accessible to clinical topographic analysis of pathology and surgery within the conventional “pons” region (e.g., modern segmental understanding of motor, reticular, vestibular, auditory, trigeminal, respiratory or cardiocirculatory functional subregions) it may take decades to extinguish its indiscriminative use as a regional descriptor for the whole rostral hindbrain.

### Recommended Brain Stem Nomenclature for Different Levels of Learning (High School, Undergraduate University/Medical School)

The clinical usage of pons and medulla oblongata is primarily based upon the external view of the human hindbrain and is commonly represented in medical student textbooks (see for example Barr's The Human Nervous System 10th edition, Kiernan and Rajakumar, 2013). Figure 5 proposes different levels of nomenclature for the hindbrain required at different levels of education. It is structured such that the lowest level of the nomenclatural understanding (high school human biology) is compatible with the more complex picture allowing a student to build on their initial simpler but already partly updated understanding of the brain stem as they progress into medical school and beyond.

## Brain Stem Nomenclature in the Terminologica Neuroanatomica

The 2017 update of Terminologica Neuroanatomica (FIPAT. Terminologica Neuroanatomica. FIPAT.library.dal.ca. Federative International Program for Anatomical Terminology. February 2017) has attempted to resolve some of the many conflicts in brain stem nomenclature. Overall, the authors have done a fine job of producing a modern nomenclature plan. However, the thickets of nomenclature are dense and challenging and there are many historical hangovers to be dealt with. From the point of view of this paper the best news is that the trochlear nucleus has been moved from the midbrain to the hindbrain. However, a number of rostral hindbrain (isthmic) structures have been unfortunately left in the midbrain. They include the cuneiform nucleus, the parabigeminal nucleus, the caudal linear nucleus, pedunculopontine tegmental nucleus (now properly called peduncular tegmental nucleus because it is nowhere near the pons), and the dorsal raphe nucleus. The latter needs explanation because a small rostral part does invade the midbrain, while the main nucleus stays in the isthmus. The interpeduncular nucleus is also included in the midbrain even though it belongs to r1.

On the rostral side of the midbrain there are some nuclei which should have been moved to the caudal diencephalon, such as the parvocellular red nucleus.

## Further Possible Changes to Traditional Names of Brain Stem Nuclei

In addition to the major nomenclatural issues described above, contemporary research points to the need for recognition of previously unrecognized features of a number of other groups of brain stem nuclei. These nuclei belong to the interpeduncular group, the precerebellar nuclei, the reticular and tegmental nuclei, and the monoaminergic nuclei of the hindbrain.

**The Location of Parts of the Interpeduncular Nucleus**

The interpeduncular nucleus (IP) occupies a subpial ventral median position associated to the hindbrain part of the interpeduncular fossa (ipf; see Figure 3 and note the classic literature often wrongly ascribed the interpeduncular fossa to the midbrain or even to the diencephalon). The IP is a bilaterally symmetrical complex of diverse subnuclei arranged anteroposteriorly and mediolaterally. The IP receives bilateral forebrain input via the habenulo-interpeduncular tract (facsiculus retroflexus) of both sides. A small rostral part of the interpeduncular nuclear complex has been experimentally demonstrated to originate from the isthmus (Lorente-Cánovas et al., 2012). This represents the prodromal (rostralmost) interpeduncular subnucleus. Caudal to this unit the interpeduncular nucleus has two main parts known as rostral IP (IPR) and caudal IP (IPC). These are located in, and originate from, rhombomere 1 (IPR, IPC; see Figure 6; Lorente-Cánovas et al., 2012).

### Precerebellar Nuclei

The precerebellar nuclei are a set of neuronal populations that generally originate from the hindbrain rhombic lip, variously migrate tangentially to diverse dorsoventral sites within a variety of hindbrain rhombomeres, and project excitatory mossy fiber input into the cerebellum, mostly contralaterally. The list of such populations includes the basilar pontine nuclei and the reticulotegmental nucleus within r3 to r4, the lateral reticular nuclei, some reticular, trigeminal and vestibular cells, and the external cuneate nucleus. The prepositus hypoglossi nucleus, the intercalated nucleus, and the nucleus of Roller (both medullary) might extend this list. The inferior olive also may be regarded as precerebellar in that sense, but it differs in that its projection ends as climbing fibers within the cerebellum, whereas the others end as mossy fibers. Finally, two previously overlooked hindbrain cell groups have been recently shown to project to the cerebellum. They are the linear nucleus and the interfascicular trigeminal nucleus.

### The Linear Nucleus—An Extension of the Lateral Reticular Nucleus

In 2009, Fu et al., showed that a dorsal extension of the lateral reticular nucleus, which they named the linear nucleus, projects to the cerebellum. This nucleus appears to be a constant feature of mammalian brains. However, it should be recognized that the first description of the nucleus, and the original application of the name linear, must be credited to Cajal (Ramon y Cajal, 1904/1995), who described it as forming a part of the lateral reticular nucleus. A segmental analysis of this nucleus in the mouse has recently been completed by Martinez-de-la-Torre et al. (2018).

### The Interfascicular Trigeminal Nucleus

This nucleus had previously been named the tensor tympani part of the motor trigeminal nucleus in rodent brain atlases (Franklin and Paxinos, 2005; Paxinos and Watson, 2007), because it was thought to be a subset of small motor neurons of the motor trigeminal nucleus innervating the tensor tympani muscle. However, the neurons forming the interfascicular trigeminal nucleus were labeled following injection of retrograde tracer in the cerebellum, and the labeled neurons were found to be choline acetyltransferase (ChAT) negative, proving that they are not motor neurons (Fu et al., 2012). In addition, the cells of the interfascicular trigeminal nucleus are strongly labeled in mice via *Wnt1Cre* and *Atoh1CreER* lineage fate mapping—a feature common to the major precerebellar nuclei that arise from the rhombic lip and that issue mossy fibers (Fu et al., 2011, 2012).

### Reticular and Tegmental Nuclei of the Brain Stem

Many nuclei in the brain stem that are not directly associated with the cranial nerves or the cerebellum have been labeled as reticular or tegmental nuclei. In the past the reticular nuclei were considered to form a heterogeneous functional group which was divided mainly into pontine and medullary reticular formation domains. This simplistic concept has been abandoned now in favor of a separate consideration of individually named reticular nuclei or cell groups, ascribed if possible to specific rhombomeres, or to rhombencephalic subregions (prepontine, pontine, retropontine, medullary). Unfortunately, some nuclei that have retained the name “reticular” belong to entirely different molecular and functional entities. These include the reticulotegmental and lateral reticular nuclei, which are both precerebellar nuclei. An associated problem is the widespread use of the imprecise term ‘ascending reticular activating system.’ This usage derives from the work of Moruzzi and Magoun (1949) who famously showed that ascending pathways from the brain stem caused the cerebrum to become alert; they assumed that the brain stem nuclei that gave rise to the ascending activating pathways must reside in the so-called reticular core of the brain stem. This proved to be incorrect, since the hindbrain cell groups that promote wakefulness do not belong to the group of identified reticular nuclei: a series of elegant studies by the Saper group (see Saper et al., 2001) have shown that the hindbrain nuclei that promote wakefulness are the locus coeruleus, the raphe nuclei, and the major forebrain and hindbrain cholinergic nuclei—none of which should be considered to belong to the reticular nuclei of the brain stem. Because of this, the term “ascending reticular activating system” should be replaced by the newer term “ascending arousal system.”

We wish to draw attention to significant nomenclatural issues relating to some nuclei in the reticular/tegmental group; these are the intermediate reticular zone, the retrorubral (now the retroisthmic nucleus), the pedunculotegmental nucleus, and the nucleus incertus.

### The Intermediate Reticular Zone

In the hindbrain the large cell (gigantocellular) reticular nuclei are medially placed and the small celled (parvicellular) reticular nuclei are laterally placed. The narrow region between these two large groups can be called the intermediate reticular nucleus (IRt).The intermediate reticular (IRt) nucleus of the rat was first recognized by Paxinos and Watson (1986) as a radial zone between the gigantocellular and parvicellular reticular nuclei which is slightly more reactive for AChE than the adjacent zones. Many peptidergic neurons tend to concentrate there (review in Puelles, 2013). This zone seems to lie next (just lateral) to the plane separating the derivatives of the alar and basal plates, which roughly extends radially from the sulcus limitans in the floor of the fourth ventricle to the pial surface of the brain stem where the vagal and glossopharyngeal rootlets emerge (Martinez-de-la-Torre et al., 2018; Puelles et al., 2018). Within the caudal part of the IRt are located the ambiguus and retroambiguus nuclei, the Botzinger (respiratory) nuclei, and the NA1 noradrenaline cell group.

### Retrorubral Nucleus

Two structures in the brain stem have been given the name “retrorubral”—the retrorubral dopaminergic field (A8 dopamine cell group) which lies selectively in m2 (Puelles et al., 2012a) and the retrorubral tegmental or reticular nucleus, which is located r1. Unfortunately, many papers confuse these two structures and the hindbrain retrorubral nucleus sometimes is described as containing dopamine neurons (probably this error relates to the observed existence of such neurons in the *isthmic* tegmentum; Puelles et al., 2012a). To avoid this confusion, Paxinos and Watson (2014) renamed the retrorubral nucleus as the “retroisthmic nucleus” since it lies immediately caudal to the caudal boundary of the isthmus. The retroisthmic nucleus is therefore defined as an area in rhombomere 1 between the pedunculotegmental nucleus medially, and the lateral lemniscus and its nuclei laterally. Rostrodorsal to it appears the microcellular tegmental nucleus of the isthmus, and rostral to it is the caudal (isthmic) pole of the substantia nigra.

### Pedunculotegmental Nucleus

The pedunculotegmental nucleus (PTg) is a prominent cholinergic (and NOS positive) cell group in r1, within the rostral hindbrain of the human, monkey, rat, and mouse. Paxinos and Watson (2006) and Puelles et al. (2007) renamed the pedunculopontine tegmental nucleus as the pedunculotegmental nucleus (PTg), because it is not a pontine structure and clearly, lying in r1, it has no close topographical relationship to the pontine nuclei in r3 and r4. It is one of many prepontine nuclei given a ‘pontine’ suffix simply because they lie in the area covered by the rostrally expanded pons in the human brain.

In the human and in the rhesus monkey, the PTg has been described as having a compact cholinergic part (pars compacta) and a diffuse non-cholinergic part (pars dissipata). In rodents, however, Swanson (1992) and Paxinos and Watson (2006) named the non-cholinergic area found lateral to PTg as the retrorubral nucleus. The retrorubral nucleus has never been recognized in primates. Paxinos and Watson (2006) concluded that the retrorubral nucleus of the rodent is, in fact, the homolog of the PTg pars dissipata of primates. A study of AChE sections of human, monkey and rat brains confirms that the PTg in all three species is strongly AChE positive in cells and neuropil. Furthermore, the area immediately lateral to PTg (the primate pars dissipata and the rodent retrorubral nucleus) in all three species is only lightly stained for AChE.

### The Incertus Nucleus

The identity of the incertus nucleus has been questioned since it was originally named by Streeter (1903). The area named by Streeter was quite extensive and includes areas not currently thought to relate to the true incertus nucleus. The current view is that the incertus nucleus lies close to the ependyma of the fourth ventricle, medial and ventral to the posterodorsal tegmental nucleus (PDTg, which lies within basal r2), close to the locus coeruleus in the rat (which lies in lateral basal r1), and consists of a medial compact part and a lateral diffuse part. The two parts of the incertus nucleus were given different names in the influential rabbit brain atlas of Meessen and Olszewski (1949). Meessen and Olzewski named the compact part as ‘nucleus O of the central gray,’ and called the diffuse part ‘the alpha part of the central gray.’ In a series of editions of the widely cited rat brain atlas (Paxinos and Watson, 1986, 1997, 1998, 2005, 2006, 2014), the authors continued to use the Meessen and Olzewski terminology. However, because the extensive recent experimental literature on the incertus nucleus has not adopted the Meessen and Olzewski nomenclature (e.g., Goto et al., 2001; Olucha-Bordonau et al., 2003; Ma et al., 2009), we feel it is time to abandon the Meessen and Olzewski terms (nucleus O and alpha parts of the central gray) in favor of the accepted modern names for the compact and diffuse parts of the incertus nucleus.

### Monoaminergic Nuclei in the Brain Stem

Monoamine groups in the brain stem were first demonstrated by Dahlström and Fuxe (1964) using the method of formalin vapor-induced fluorescence. The original description of the anatomy of these groups was further developed by Fuxe et al. (1970) and Hökfelt et al. (1974) and many subsequent publications by this group. The fluorescent cell groups were originally given arbitrary names (A1, A2 etc. and B1, B2 etc.), and these alphanumeric titles do not provide information concerning the function of the different groups. Because of this, we recommend following the nomenclature adopted by Paxinos et al. (2012) in their atlas of the marmoset brain, and subsequently adopted in atlases of the rat brain (Paxinos and Watson, 2014) and mouse brain (Paxinos and Franklin, 2013). Paxinos et al. (2012) named dopamine groups with the prefix DA, noradrenalin groups with the prefix NA, and adrenaline groups with the prefix Ad. However, we have retained the name of locus coeruleus for the previously named A6 group, and the name supralemniscal nucleus for the B9 serotonin group. Similarly, we have retained the names retrorubral field (RRF), substantia nigra compact part (SNC), and ventral tegmental area (VTA) for the dopamine groups previously defined as A8, A9, and A10 (Paxinos et al., 2012).

### Many Previously Unrecognized Brain Stem Nuclei Have Appeared in Atlases Since 1982

The various editions of the Paxinos and Watson rat brain atlas since 1982 have identified and named many brain stem nuclei that had not been defined in previous atlases. Many of these newly identified nuclei have since been identified in atlases of the brains of the mouse (Paxinos and Franklin, 2013), marmoset (Paxinos et al., 2012), rhesus monkey (Paxinos et al., 2009), and human (Paxinos et al., 2018). These newly identified nuclei include the rhabdoid nucleus, the interstitial nucleus of the superior cerebellar peduncle, and the trigeminosolitary transition zone.

## The Use of Eponyms

Over the last 50 years there has been a sensible push to reduce the number of eponyms used in describing neuroanatomical features, and there is a logical argument to remove them all. However, we agree with Paxinos and Watson (2014) that there is no real prospect of expunging a small number of famous and popular eponyms in relation to the brain stem, and we should simply accept their existence. We would therefore retain Barrington's nucleus, the nucleus of Darkschewitsch, the nucleus of Roller, the interstitial nucleus of Cajal, the Edinger-Westphal nucleus, and the cap of Kooy (inferior olive). We observe that in recent years we have also been forced to accept one new eponym—that of Botzinger.

## Recommendations

1. Abandon the subdivision of the hindbrain into “pons” and “medulla.”
2. Restrict the use of the term ‘pons’ to refer to the nuclei and fiber bundles of the basilar pontine formation.
3. Recognize the isthmus (rhombomere 0) as the first segment of the hindbrain.
4. Recognize that the cerebellum is a derivative of the rostral prepontine hindbrain.
5. Recognize that the posterior commissure and associated nuclei, the nucleus of Darkshewitsch, the interstitial nucleus of Cajal, and the rostral part of the red nucleus belong to the caudal diencephalon and not to the midbrain.
6. Consider the evidence for including the midbrain in the forebrain on genoarchitectural grounds, which would have the effect of making the old term “brain stem” synonymous with the hindbrain.
7. Adopt a modern functional and segmental nomenclature for the classification of the monoamine cell groups of the brain stem (see Alonso et al., 2012 for serotonergic cell groups of the hindbrain raphe).

## Author Contributions

LP was primarily responsible for the direction of this research. CW was responsible for the design of the paper. All three authors contributed equally to the writing of this paper.

### Conflict of Interest Statement

The authors declare that the research was conducted in the absence of any commercial or financial relationships that could be construed as a potential conflict of interest.
